# Relationship between Eating Habits and 4-Nonylphenol Concentration in Breast Milk of Women in Slovakia

**DOI:** 10.3390/life13122361

**Published:** 2023-12-18

**Authors:** Adriana Repková, Csilla Mišľanová, Janka Hrabčáková, Marián Masár, Zuzana Slezáková, Lukáš Žemlička, Martina Valachovičová

**Affiliations:** 1 Department of Midwifery, Faculty of Nursing and Professional Health Studies, Slovak Medical University, 833 03 Bratislava, Slovakia; adriana.repkova@szu.sk (A.R.); janka.hrabcakova@szu.sk (J.H.); 2 Institute of Nutrition, Faculty of Nursing and Professional Health Studies, Slovak Medical University, 833 03 Bratislava, Slovakia; martina.valachovicova@szu.sk; 3 Department of Analytical Chemistry, Faculty of Natural Sciences, Comenius University, 842 15 Bratislava, Slovakia; marian.masar@uniba.sk; 4 Department of Nursing, Faculty of Nursing and Professional Health Studies, Slovak Medical University, 833 03 Bratislava, Slovakia; zuzana.slezakova@szu.sk; 5 Department of Nutrition and Food Quality Assessment, Institute of Food Science and Nutrition, Faculty of Chemical and Food Technology STU, 812 37 Bratislava, Slovakia; lukas.zemlicka@stuba.sk

**Keywords:** 4-nonylphenol, breast milk, HPLC-SPE analysis, breastfeeding, lifestyle

## Abstract

4-Nonylphenol belongs to the alkylphenol group of chemicals, and its high occurrence in the environment can cause an adverse effect on human health. Breast milk can serve as a marker to take measure of human exposure to these chemicals through different routes of exposure. In this work, the influence of selected factors (the kind of water drank by the mothers; the consumption of fish, pork, and beef; wearing gloves; using nail polish, gel nails, vitamins, and medication) on the concentration on 4-nonylphenol in 89 breast milk samples was studied. The concentrations of nonylphenol in breast milk were determined by HPLC with fluorescence detection. The lowest and highest concentrations of 4-nonylphenol in breast milk were 0.97 ng/mL and 4.37 ng/mL, respectively. Statistical significance was observed for the consumption of pork (*p* = 0.048) and fish (0.041) in relation to the 4-nonylphenol concentration. Certain parameters (use of gel nails, beef consumption, and vitamin supplementation) were at the border of statistical significance (*p* = 0.06). Other parameters did not show any statistical significance. The results showed that breast milk in Slovakia does not contain a harmful dose of 4-nonylphenol and does not cause health problems. But it is necessary to continue this research and perform extended screening on a larger number of samples.

## 1. Introduction

Alkylphenols belong to a group of endocrine disruptors (ED) and xenoestrogen, which are exogenous substances disrupting the endocrine system. They can disrupt the hormonal structure of the body and have adverse effects on human health. The growing attention regarding the health of the next generation is devoted to the effect of endocrine-disrupting chemicals on human health [1,2,3,4]. Nonylphenols are used for the production of nonylphenol polyethoxylates (NPEOs), the most often used nonionic surfactans in industrial and household products, personal care products, detergents, emulsifiers, additives in cosmetic products, water-based paints, textiles, the production of plastics, resins, paints, cleaners, pesticides, etc. [5]. NPEOs are also used in “Industrial and Institutional cleaners” (I&I), which are the second largest cleaning product category after household detergents. We can encounter these cleaning processes, for example, in hotels, restaurants, schools, office complexes, hospitals, etc. These cleaners are more aggressive and more alkaline than household and consumer products [6].

Nonylphenol is a mixture of different isomers (ortho-, meta-, or para-), which are located by the arrangement of the carbon chain, and the most common isomer is the para isomer (4-NP). 4-NP is a derivative of phenol, where a carbon chain with nine carbon atoms is attached to its aromatic ring. It is a potential threat to the environment because it is toxic to aquatic organisms and can have harmful effects on the ecosystems of waterways and seas [7]. In some legislation [8], measures were taken to limit the use of NP and its compounds in some products and industrial processes in order to minimize the risks associated with this substance.

NP and NPEOs are regulated in the European Union under Directive 2003/53/EC, which was adopted on 18 June 2003 and became effective on 17 January 2005 [9]. This directive sets restrictions on the use of these substances in various products. According to this directive, it is prohibited to market and use textile products that contain NP and NPEOs in concentrations higher than 0.1% by weight. In addition, the use of these substances in washing preparations, detergents, and cleaning agents is prohibited if it leads to the emulsification of hazardous substances in water. However, it is possible that the rules and regulations can be changed and supplemented. Therefore, it is important to follow the current information and updates from the European Union and important countries regarding the use of NP [10,11,12].

Recently, the high occurrence of persistent endocrine disruptors in the environment has caused growing concern about their impact on human health. Infants are particularly sensitive to hormonal influences from environmental chemicals. Due to their presence in human milk, concerns for mothers and children´s health are increasing [13,14]. Due to its lipophilic character, ED can be accumulated in maternal body fat, can pass through the placental barrier along with residual contaminants, and can be transferred from plasma into breast milk during lactation period [5,15].

Breast milk is among the body fluids most suitable for monitoring the effect of alkylphenol exposure on both mother and child [16]. Breast milk is a complex and variable fluid that evolved to nourish the infant and protect it from disease while its immune system continues to develop. The composition of human breast milk varies depending on many factors that correspond to the needs of the child according to its age and other factors [17,18,19]. Human breast milk is an irreplaceable form of nutrition for newborns, and its consumption provides important benefits for the newborn’s immune system, growth, and psychological development. Breastfed babies have a more stable and less diverse intestinal microflora than formula-fed babies, but they have more than twice the number of bacterial cells. This may be partly due to changes at the level of the intestinal mucosa due to bioactive substances in breast milk. Moreover, breastfeeding reduced the risk of developing different allergies, infections, and other chronic diseases and decreased mortality and morbidity in infants as well as the occurrence of cancer and osteoporosis in mothers [20]. However, breastfeeding, which is the only source of nutrition for infants and babies, can also be a route of contamination for children. Breast milk can serve as a marker to measure human exposure to these chemicals through different routes of exposure. The main routes of human exposure are through chemicals’ residue in food, water, and air [6,21,22,23]. 4-NP is excreted by the organism, and, therefore, its concentration in breast milk can be affected by various factors such as diet, environment, and individual differences (e.g., in the rate of metabolism) between people. However, it is important to emphasize that the presence of NP in breast milk varies between regions and women. It is important to remember that the benefits of breastfeeding for the baby outweigh the potential risks associated with exposure to 4-NP.

4-Nonylphenol is a chemical substance that has been banned in the EU for household use since 2012 and is only allowed for professional cleaning. In our study, we monitored whether nursing mothers come into contact with this substance by using household cleaning products and cosmetics as well as through their food consumption. To date, no studies have monitored the relationship between these parameters and nonylphenol concentration in breast milk in Slovak nursing women. The aim of this pilot study was to investigate the selected parameters of eating habits and 4-nonylphenol concentration. The high-performance liquid chromatography–fluorescence detection method with Chromabond C18ec SPE sorbent was used for the determination of 4-nonylphenol in breast milk samples. In the future, we would like to introduce the method into routine practice.

## 2. Materials and Methods

### 2.1. Characteristics of Mothers and Children

Initially, 110 nursing mothers were approached, of which 89 mothers willing to collect breast milk were included in the study, while 21 mothers refused to provide a breast milk sample without giving a reason. Mothers had university degree (89.90%) or secondary (10.10%) education. The characteristics of mothers and children are presented in Table 1. Nursing mothers who were expected to spontaneously give birth without complications were included in the study. All participants came from Bratislava and its surroundings, and they were subjectively healthy and willing to participate in the trial.

### 2.2. Milk Sample Collection and Storage

The nursing mothers were from the gynecology clinic of the University Hospital in Bratislava. Each nursing mother from study provided 15 mL of freshly sucked breast milk at the gynecologic clinic. Milk samples were collected after 2–4 weeks by manual expression in chemical-free glass tubes. Samples were taken twice, in the first month and in the fourth month of breastfeeding in the morning between 8 and 10 a.m. The samples were transported on dry ice to the laboratory and stored at −20 °C until analysis. Plastics were excluded during analysis to avoid further contamination. Trained nurse from the gynecology clinic was responsible for transporting the samples. 4-nonylphenol concentrations in breast milk were determined by the HPLC method with fluorescence detection.

### 2.3. Reagents and Chemicals

4-nonylphenol and 4-octylphenol were supplied by Sigma Aldrich (Steinheim, Germany). Formic acid (98%), methanol (HPLC gradient grade), acetonitrile (HPLC gradient grade), and water were purchased from Merck (Darmstad, Germany).

The Chromabond C18ec (100 mg/1 mL, 45 µm) was obtained from Macherey-Nagel (Germany). SPE experiments were performed using a Visiprep SPE Vacuum Manifold DL (Sigma Aldrich, Germany). After elution, samples were evaporated under nitrogen, and residues were dissolved in mobile phase.

### 2.4. Sample Pretreatment

Considering the complexity of the breast milk samples and the low concentration levels of nonylphenol (µg/L), HPLC analysis of these substances requires sample pretreatment. During this phase, together with the concentration of the analyte, all potential interferences that can affect the sensitivity and accuracy of the method are eliminated. The most frequently used technique for sample treatment is solid phase extraction (SPE) based on the affinity of analytes to the sorbent [13,24,25,26,27]. Different types of SPE sorbents (C18, Oasis HLB, Strata-X, etc.) are available for extraction of target analytes. Breast milk samples were first subjected to liquid–liquid extraction, and then each extract was processed by SPE. Firstly, breast milk samples were incubated at 37 °C, sonicated in order to disrupt the fat milk layers, and then centrifuged at 15,000× *g* for 15 min. Subsequently, 0.5 mL of 0.1% (*v*/*v*) formic acid in methanol was added to 0.5 mL of skimmed milk, and sample was vortexed for 3 min and centrifuged at 14,000 for 10 min. Later, 0.5 mL of methanol was added to supernatant, and sample was centrifuged for 10 min. Then, 50 μL of 4-octylphenol as internal standard was added to supernatant (450 μL) to obtain the final volume of 0.5 mL, which was further pretreated by solid-phase extraction. Individual steps of SPE procedure are summarized in Table 2.

### 2.5. HPLC Analysis

High-performance liquid chromatography (HPLC) with fluorescence detection was used for determination of 4-nonylphenol in breast milk. All analyses were performed using the HPLC (HP 1200, Agilent, Munich, Germany) chromatograph with fluorescence detection. Chromatographic separation was achieved on a Nucleodur C18ec column (150 × 4.6 mm, 5 μm, Macherey-Nagel, Germany) with a precolumn C18 (10 × 4.6 mm, 5 μm). Mobile phase consisted of acetonitrile (A) and water (B). The linear gradient was as follows: 0–3.9 min 30% B; 4 min 70% B; 4.2–10 min 100% B; 10.1 min 30% B; 13.5 min stop. Flow rate of mobile phase was 1 mL/min. Sample injected volume was 25 µL.

### 2.6. Validation

In general, a validated analytical method gives reliable and reproducible results and clearly defined variable parameters (FDA, 2001). The present method was validated according to linearity, extraction recovery, limit of detection, and quantification.

#### 2.6.1. Linearity

Seven calibration standards were used to determine linearity on 10 separate days, and each calibration standard was analyzed three times. Calibration curves were prepared from 0.5 to 100 ng/mL for 4-nonylphenol (NP) in breast milk and 10 ng/mL for 4-octylphenol as internal standard. Coefficients of determination (R^2^) obtained by regression analysis were higher than 0.99.

#### 2.6.2. Extraction Recovery

The extraction recovery of 4-nonylphenol were determined at three concentration levels (1, 10, and 50 ng/mL) of calibration sample in milk. The recoveries of analyte were calculated by comparing the peak area from milk sample spiked before extraction with milk sample spiked after extraction, which represented 100% recovery. Extraction recoveries for 4-NP were 101.5% ± 6.2 for 1 ng/mL; 83.5% ± 4.3 for 10 ng/mL; 80.5 ± 2.6 for 50 ng/mL. Recovery for 4-OP was 85.1% ± 2.9.

#### 2.6.3. Limit of Detection and Quantification

The limit of detection (LOD) for extracted milk samples was calculated based on a signal-to-noise ratio of three for the analytical peak area of the maximum fluctuation of the noise for 10 blank milk samples and sample spiked to known analyte concentrations. LOD for 4-NP was 0.27 ng/mL. The limit of quantitation was defined as the lowest concentration of the calibration standard for which an acceptable accuracy within ±15% and a precision less than 15% were obtained. The signal intensity at this low concentration was at least 10 times higher than the signal intensity of the blank milk. LOQ for 4-NP was 0.91 ng/mL.

### 2.7. Questionnaire

This study used a self-constructed questionnaire that contained 50 questions. The questions dealt with the education and accommodation of the nursing mother, the health status of the baby and nursing mother, socioeconomic status, use of cosmetics and cleaning products, and eating habits.

### 2.8. Ethics Committee and Informed Consent

The Research Ethics Committee of the Slovak Health University in Bratislava approved the project, in accordance with the rules and guidelines for human research, before the start of research in 2022 (no. 13/2022). Participants were informed about the development of the research, and those who agreed to participate signed two copies of an informed consent form, one of which was given to the nursing mother.

### 2.9. Statistical Analysis

Data were analyzed using SPSS version 19 (Statistical Package for the Social Science, Chicago, IL, USA) for Windows. The normality of data distribution was tested using Shapiro–Wilk test. Data were not normally distributed. Therefore, Spearman´s rank correlation was used to examine the relationship between NP concentrations and socio-demographic and maternal parameters. Non-parametric tests (Mann–Whitney and Kruskal–Wallis) were used to determine differences in NP concentrations between different groups. The significance level was set at *p* ≤ 0.05.

## 3. Results

On our questionnaire, we asked the birth weight, length, and Apgar score of the child. The Apgar score is a simple method that is used to evaluate the health status of a newborn in the first minutes of life (Table 1). It is determined concerning five criteria—heart rate, breathing, muscle tone, response to irritation, and skin color. This score provides a quick and objective value of the newborn’s health status. We found that the measured concentrations of nonylphenol in breast milk do not affect any of the given parameters in the newborn, for example, the birth weight, the birth length, or the Apgar score of the child. In the questionnaire, we identified what kind of water mothers drank (Table 3). All food came from local stores and/or sources. Each nursing mother included in the study consumed homemade food, without any fast food or highly processed food products. During breastfeeding, they ate meat three times per week and fish once a week.

The lowest and highest concentrations of NP in breast milk were 0.97 ng/mL and 4.37 ng/mL, respectively. The average concentration ± STD was 3.40 ± 0.27 ng/mL.

Higher concentrations were observed in mothers consuming pork (3.31 ng/mL) and fish (3.14 ng/mL) compared to non-consumers (3.03 and 2.89 ng/mL, respectively). This parameter was statistically significant for pork consumption (*p* = 0.048) as well as for fish consumption (*p* = 0.041), in relation to the 4-NP concentration. The results are presented in Table 4.

In addition, it was shown that mothers with gel nails have higher concentration of 4-NP (*p* = 0.06). At the border of statistical significance was beef consumption (*p* = 0.06) and vitamin supplementation (*p* = 0.06). The findings are presented in Table 5 and Table 6. The other monitored factors did not have a significant influence on nonylphenol values.

In Table 5 and Table 6, we can see the influence of wearing gloves and using nail polish and gel nails as well as using vitamins and medicines, respectively, on the nonylphenol concentration in nursing mothers.

## 4. Discussion

Breast milk remains the best source of infant nutrition, but constant surveillance is needed to keep it pure. Breastfeeding offers many advantages to neonates and infants and provides a range of short- and long-term benefits for growth, immunity, and cognitive and psychological development, as well as protection against infection, allergies, and other chronic diseases. The composition of human milk varies at different stages of lactation, distinct times of the day, during each feed, and even between breasts; it contains powerful growth- and immune-enhancing factors; and breastfeeding is considered as the best and only source of nutrition necessary for the infant during the first 6 months of life, as recommended by the WHO. Chemical contaminants in milk, if the level is high enough or if the infant is sensitive enough, interact at many possible physiological levels. Numerous studies have associated breastfeeding with potential medical and social benefits, which include decreased mortality and morbidity from infectious and other diseases, influence on brain development, increased resistance to chronic diseases in infants and a decreased incidence of cancer and osteoporosis in mothers. Some mothers avoid breastfeeding on personal or social grounds, but the reasons can be associated with complications including secretion, breast pain, engorgement, and mastitis [28]. The adverse events associated with drug exposure via lactation occur most often in neonates younger than 2 months and rarely in infants older than 6 months [29].

4-Nonylphenol (NP) is a chemical substance used as an additive in some industrial processes and products, and it can occur in the home environment. Another factor may include environmental pollution. If the nursing mother’s accommodation is near industrial zones, a waste dump, or other sources of pollution, there is a higher probability that NP can get into her body and, subsequently, into her breast milk.

In the presented study, we investigated the relationship between the 4-NP concentration in breast milk samples and various factors, such as demographic data, dietary habits, the health status of the mother and child, and the use of cleaning products and cosmetics.

We did not observe significant correlations between the 4-NP concentration and selected factors including the age of the mother, BMI, education, obstetric history, and the health status of the mother and child. Similar conclusions were reached in [30,31].

In our study, we monitored the influence of the place of residence on the NP concentration. We supposed that nursing mothers living in a village would have lower 4-NP concentrations than those living in a city. This assumption was not confirmed. Mothers living in a village had increased 4-NP concentrations, but these findings were not statistically significant. Increased values probably must be attributed to the fact that mothers can live near an industrial zone or a polluted environment in their village.

Possible ways of human exposure to 4-NP can include the consumption of food, drinking water, or contact with cleaning agents and cosmetics [32]. The consumption of freshwater and marine fish, fish oil capsules, and meat was discussed by Chen et al. [30]. The relationship between the consumption of freshwater and sea fish showed statistical significance (*p* < 0.01), while the consumption of meat did not show an association with the concentration of nonylphenol. The concentrations of alkylphenols in Taiwanese rivers and sediments were higher compared to, for example, those in Japan or Germany. The application of sewage sludge to agricultural land can cause the soil entry of alkylphenols. The persistence of alkylphenols causes entry into the food chain and accumulation in fish.

In our study, we observed statistical significance (*p* = 0.041) for fish consumption as well as for pork consumption (*p* = 0.048), in contrast to the Taiwanese study. But there were no correlations between the frequency of meat and fish consumption.

A similar study was conducted in Turkey in 2017, where the authors found that mothers who consumed fresh fish had higher concentrations of 4-nonylphenol. Also, mothers who used cleaning products had significantly higher concentrations of 4-nonylphenol in their breast milk [33]. This was not confirmed in our study.

Breast milk is the basis of the healthy development of an individual. In our study, we dealt with the occurrence of 4-nonylphenol in human milk. 4-nonylphenol is a chemical substance that was used until 2012 [8,9,10,11,12,34,35]. From that year onwards, 4-nonylphenol has only been accessible to industry. Since it is a lipophilic substance, we were interested in whether the given substance binds to breast milk and passes into the child’s organism in nursing mothers. So far, there is no study in Slovakia that would investigate the relationship between eating habits and the concentration of alkylphenols in breast milk.

The Tolerable Daily Intake (TDI) for a 5 kg body weight (bw) for 4-NP was proposed by the Danish Institute of Safety and Toxicology [36]. From the obtained 4-NP concentration, it is possible to calculate the daily intake. Using an average 4-NP concentration of 3,4 ng/mL, the daily intake for Slovak infants (with a 5 kg bw) consuming 500 mL of breast milk was calculated as 0.26 μg/kg/d. This value is much lower than those obtained in the Taiwanese study (4.47 μg/kg/d) [30], Japanese study (0.65–1.4 μg/kg/d) [37], and Italian study (3.94 μg/kg/d) [13]. In the Taiwanese study, the authors examined the dietary habits of 59 mothers. They observed a significant association (*p* < 0.05) between the frequency of cooking oil and fish oil capsule (*p* < 0.01) consumption and the OP concentration.

A similar value for the TDI as in our study (0.26 μg/kg/d) was published in a German study (0.3 μg/kg/d) [38], where NPs in 60 different food samples, including breast milk, were analyzed. It was assumed that, due to the lipophilic properties of NPs, high concentrations of NPs would be obtained in fatty food (e.g., butter, liver sausage, etc.). But high NP concentrations were also observed in nonfatty foods like fruits.

NPs, octylphenol (OP), NP monoethoxylate (NP1EO), and two octylphenol ethoxylates (OPEOs) (namely, OP1EO and OP2EO) were detected in the human breast milk of Italian women [13]. 4-NP was the contaminant found at the highest levels (32 ng/mL), about two orders of magnitude higher than OP (0.08 ng/mL), OP1EO (0.07 ng/mL), and OP2EO (0.16 ng/mL). In this study group, a positive correlation between fish consumption and the levels of 4-NP in milk was observed, in accordance with the evidence that seafood represents one of the most important sources of exposure to this group of contaminants in Italy. The high concentrations of alkylphenols can be linked to environmental contamination or to bioaccumulation in food of animal sources. On the basis of the concentrations found in the breast milk samples, a maximum 4-NP daily intake of 3.94 mg/kg/day can be calculated, which is close to the TDI of 5 mg/kg bw.

The data from our study can be considered as preliminary research to determine the presence of NPs in milk samples. The results indicate that further research would be needed to better assess neonatal exposure to environmental contaminants.

## 5. Conclusions

Human milk is one way of removing the mother’s environmental toxic chemicals’ burden. The assessment of the presence and quantity of the environmental toxic chemicals in breast milk provides information on the maternal toxic load and serves as a marker of prenatal fetal exposure to this chemical.

The Tolerable Daily Intake for Slovak infants (with a 5 kg bw) consuming 500 mL of breast milk was calculated as 0.26 μg/kg/d, and it was comparable to the value from the German study.

In this study, the relationship between eating habits and the concentration of 4-nonylphenol in breast milk was investigated. The consumption of pork and fish was statistically significant in relation to the 4-nonylphenol concentration. Parameters such as vitamin supplementation and using gel nails appeared at the border of statistical significance.

Based on the obtained results, we can evaluate that breast milk contains concentrations of nonylphenol at reference values and, thus, does not affect human health. This confirms that nonylphenol is not present in high doses in the mother’s body and, therefore, does not cause health problems, so breast milk is a safe food for the child. It is very important that such studies are carried out in order to better understand the potential risks to the health of children and mothers.

Risk management should, therefore, focus on reducing lifetime exposure, especially during the prenatal period and pregnancy, since toxic chemicals accumulate before pregnancy and are released during pregnancy and lactation.

## Figures and Tables

**Table 1:** Description of mothers and children.

Maternal Characteristics			Children’s Characteristics	
Parameter	Mean ± STD		Parameter	Mean ± STD
Age	32.29 ± 3.68		Age in months	2.42 ± 1.06
Weight (kg)	65.11 ± 12.02		Weight at birth (kg)	3.51 ± 2.12
Height (cm)	167.01 ± 5.90		Length at birth (cm)	50.24 ± 1.98
BMI	23.32 ± 4.02		Apgar score after birth	9.75 ± 0.67
First menstruation age	13.18 ± 1.38			
Number of pregnancies (%)	1	57.30		
	2	27.00		
	3	15.70		

**Table 2:** Solid-phase extraction procedure using C18ec sorbent for target analytes.

Extraction Steps	Chromabond C18ec
Conditioning	1 mL methanol 1 mL water
Sample load	0.5 L with ISTD
Cleaning	1 mL 5% methanol
Drying	Under vacuum 1–2 min
Elution of analytes	1 mL acetonitrile

**Table 3:** Statistical evaluation of effect of different kind of drinking water on 4-NP concentration in nursing mothers.

Kind of Drinking Water	n	Mean ± STD NP (ng/mL)	Std. Error Mean	95% Confidence Interval for Mean		Minimum	Maximum
				Lower bound	Upper bound		
Bottled	22	2.28 ± 0.29	0.06	2.05	2.40	0.38	1.69
Tap	53	1.16 ± 0.31	0.04	1.07	1.24	0.67	2.27
Other	14	2.08 ± 0.23	0.06	1.95	2.21	0.57	1.47
Total	89	1.15 ± 0.29	0.03	1.09	2.31	0.38	2.27

n—number of samples; NP—nonylphenol; *p* = 0.587.

**Table 4:** Statistical evaluation of effect of meat and fish consumption on 4-NP concentration in nursing mothers.

		Mean ± STD NP (ng/mL)	STD Error Mean	
Consumption of fish	n			
yes	80	3.14 ± 0.30	0.03	*p* = 0.041
no	9	2.89 ± 0.21	0.07	
Consumption of pork				
yes	60	3.31 ± 0.32	0.04	*p* = 0.048
no	29	3.03 ± 0.21	0.04	
Consumption of beef				
yes	49	3.23 ± 0.35	0.05	*p* = 0.06
no	40	2.98 ± 0.21	0.03	

n—number of samples; NP—nonylphenol.

**Table 5:** Statistical evaluation of effect of wearing gloves and using nail polish and gel nails on 4-NP concentration in nursing mothers.

Wearing Gloves for Cleaning	n	Mean ± STD NP (ng/mL)	STD Error Mean	
yes	20	2.83 ± 0.28	0.06	*p* = 0.709
no	69	2.26 ± 0.29	0.04	
Using nail polish				
yes	19	2.23 ± 0.28	0.07	*p* = 0.729
no	70	2.15 ± 0.29	0.04	
Gel nails				
yes	19	4.21 ± 0.28	0.07	*p* = 0.06
no	70	4.14 ± 0.29	0.04	

n—number of samples; NP—nonylphenol.

**Table 6:** Statistical evaluation of effect of vitamins and medication use on 4-NP concentration in nursing mothers.

Vitamin Supplements	n	Mean ± STD NP (ng/mL)	STD Error Mean	
yes	60	3.48 ± 0.29	0.04	*p* = 0.06
no	27	3.06 ± 0.29	0.06	
Medication				
yes	23	2.94 ± 0.34	0.07	*p* = 0.093
no	65	2.72 ± 0.27	0.03	

n—number of samples; NP—nonylphenol.

## Data Availability

Data collected during this study are available on request from the corresponding author.

## References

[1] Raecker T., Thiele B., Boehme R.M., Guenther K. (2011). Endocrine disrupting nonyl- and octylphenol in infant food in Germany: Considerable daily intake of nonylphenol for babies. Chemosphere.

[2] Acir I.-H., Guenther K. (2018). Endocrine-disrupting metabolites of alkylphenol ethoxylates—A critical review of analytical methods, environmental occurrences, toxicity, and regulation. Sci. Total Environ..

[3] Yi B., Kim C., Park M., Han Y., Park J.Y., Yang M. (2013). Association between Endocrine Disrupting Phenols in Colostrums and Maternal and Infant Health. Int. J. Endocrin..

[4] Günther K., Racker T., Bohme R. (2017). An Isomer-Specific Approach to Endocrine-Disrupting Nonylphenol in Infant Food. J. Agric. Food Chem..

[5] Osimitza T.G., Droegea W., Driverb J.H. (2015). Human Risk Assessment for Nonylphenol. Hum. Ecol. Risk Assess. Inter. J..

[6] Zoller U. (2008). Handbook of Detergents, Part E: Applications.

[7] Soares A., Guieysse B., Jefferson B., Cartmell E., Lester J.N. (2008). Nonylphenol in the envi-ronment: A critical review on occurrence, fate, toxicity and treatment in wastewaters. Environ. Int..

[8] European Commission (2016). Commission Regulation (EU) 2016/26 of 13 January 2016 Amending Annex XVII to Regulation of the European Parliament and of the Council (EC) No. 1907/2006 on the Registration, Evaluation, Authorization and Restriction of Chemicals (“REACH”) as Regards Nonylphenol Ethoxylates.

[9] European Parliament (2003). Directive 2003/53/EC of the European Parliament and of the Council of 18 June 2003 Amending for the 26th Time Council Directive 76/769/EEC Relating to Restrictions on the Marketing and Use of Certain Dangerous Substances and Preparations (Nonylphenol, Nonylphenol Ethoxylate and Cement): DIRECTIVE 2003/53/EC.

[10] European Union (2008). Environmental Quality Standards Directive.

[11] European Commission (2009). Tris(nonylphenyl)phosphite_Cosmetic Ingredient Database (CosIng). https://ec.europa.eu/growth/toolsdatabases/cosing/index.cfm?fuseaction=search.details_v2&id=38769.web.

[12] European Commission (2002). European Union Risk Assessment Report: 4-Nonylphenol (Branched) and Nonylphenol: EUR 20387 EN.

[13] Ademollo N., Ferrara F., Delise M., Fabietti F., Funari E. (2008). Nonylphenol and octylphenol in human breast milk. Environ. Int..

[14] Hudson R.E., Metz T.D., Ward R.M., McKnite A.M., Enioutina E.Y., Sherwin C.M., Watt K.M., Job K.M. (2023). Drug exposure during pregnancy: Current understanding and approaches to measure maternal-fetal drug exposure. Front. Pharmacol..

[15] Huang Y.-F., Wang P.-W., Huang L.-W., Yang S.-H., Chiu H.-H., Chen M.-L. (2014). Nonylphenol in pregnant women and their matching fetuses: Placental transfer and potential risks o infants. Environ. Res..

[16] Ringbeck B., Bury D., Lee I., Lee G., Alakeel R., Alrashed M., Tosepu R., Jayadipraja E.A., Tantrakarnapa K., Kliengchuay W. (2022). Biomarker-Determined Nonylphenol Exposure and Associated Risks in Children of Thailand, Indonesia, and Saudi Arabia. Environ. Sci. Technol..

[17] Ballard O., Morrow A.L. (2013). Human milk composition: Nutrients and bioactive factors. Pediatr. Clin. N. Am..

[18] Lu Y.Y., Chen M.L., Sung F.C., Paulus S.-G.W., Mao I.F. (2007). Daily intake of 4- nonylphenol in Taiwanese. Environ. Int..

[19] Nickerson K. (2006). Environmental contaminants in breast milk. J. Midwifery Womens Health.

[20] Noorimotlagh Z., Haghighi N.J., Ahmadimoghadam M., Rahim F. (2017). An updated systematic review on the possible effect of nonylphenol on male fertility. Environ. Sci. Pollut. Res..

[21] Careghini A., Mastorgio A.F., Saponaro S., Sezenna E. (2015). Bisphenol A, nonylphenols, benzophenones, and benzotriazoles in soils, groundwater, surface water, sediments, and food: A review. Environ. Sci. Pollut. Res..

[22] Gyllenhammar I., Glynn A., Darnerud P.O., Lignell S., van Delft R., Aune M. (2012). Nonylphenol and bisphenol A in Swedish food and exposure in Swedish nursing women. Env. Int..

[23] Hong Y., Feng C., Yan Z., Wang Y., Liu D., Liao W., Bai1 Y. (2020). Nonylphenol occurrence, distribution, toxicity and analytical methods in freshwater. Environ. Chem. Lett..

[24] Azzouz A., Rascón A.J., Ballesteros E. (2016). Simultaneous determination of parabens, alkylphenols, phenylphenols, bisphenol A and triclosan in human urine, blood andbreast milk by continuous solid-phase extraction and gas chromatography–mass spectrometry. J. Pharm. Biomed. Anal..

[25] Caban M., Stepnowski P. (2020). The quantification of bisphenols and their analogues in wastewaters and surface water by an improved solid-phase extraction gas chromatography/mass spectrometry method. Environ. Sci. Polutt..

[26] Lee S.M., Cheong D., Kim M., Kim Y.-S. (2023). Analysis of Endocrine Disrupting Nonylphenols in Foods by Gas Chromatography-Mass Spectrometry. Foods.

[27] Núňez L., Turiel E., Tadeo J.L. (2007). Determination of nonylphenol and nonylphenol ethoxylates in environmental solid samples by ultrasonic-assisted extraction and high performance liquid chromatography-fluorescence detection. J. Chromatogr. A..

[28] Stuebe A. (2009). The risks of not breastfeeding for mothers and infants. Rev. Obstet. Gynecol..

[29] Anderson P.O., Pochop S.L., Manoguerra A.S. (2003). Adverse drug reaction in breastfed infants: Less than imagined. Clin. Pediatr..

[30] Chen G.-W., Ding W.-H., Ku H.-Y., Chao H.-R., Chen H.-Y., Huang M.-C., Wang S.-L. (2010). Alkylphenols in human milk and their relations to dietary habits in central Taiwan. Food Chem. Toxicol..

[31] Chen M.L., Lee W.P., Chung H.Y., Guo B.R., Mao I.F. (2005). Biomonitoring of alkylphenols exposure for textile and housekeeping workers. Int. J. Environ. Anal. Chem..

[32] Votavová L., Dobiáš J., Voldřich M., Čížková H. (2009). Migration of nonylphenols from polymer packaging materials into food. Czech J. Food Sci..

[33] Sise S., Uguz U. (2017). Nonylphenol in Human Breast Milk in Relation to Sociodemo-graphic Variables, Diet, Obstetrics Histories and Lifestyle Habits in a Turkish Population. Iran. J. Public. Health.

[34] Kim S.H., Nam K.H., Hwang K.A., Choi K.C. (2016). Influence of hexabromocyclododecane and 4-nonylphenol on the regulation of cell growth, apoptosis and migration in prostatic cancer cells. Toxicol. Vitr..

[35] Schrenk D., Bignami M., Bodin L., Chipman J.K., del Mazo C., Grasl-Kraupp B., Hogstrand C., Hoogenboom L., Leblanc J.-C., EFSA Panel on Contaminants in the Food Chain (CONTAM) (2021). Update of the risk assessment of hexabromocyclododecanes (HBCDDs) in food. EFSA J..

[36] Nielsen E., Østergaard G., Thorup I., Ladefoged O., Jelnes O., Jelnes J.E. (2000). Toxicological Evaluation and Limit Values for Nonylphenol, Nonylphenol Ethoxylates, Tricresyl, Phosphates and Benzoic Acid.

[37] Otaka H., Yasuhara A., Morita M. (2003). Determination of bisphenol A and 4-nonylphenol in human milk using alkaline digestion and cleanup by solid-phase extraction. Anal. Sci..

[38] Guenther K., Heinke V., Thiele B., Kleist E., Prast H., Raecker T. (2002). Endocrine Disrupting Nonylphenols are ubiquitous in food. Environ. Sci. Technol..

